# The potential of serum fetal DNA for early diagnosis of gestational trophoblastic disease

**DOI:** 10.4274/tjod.galenos.2019.54815

**Published:** 2020-02-28

**Authors:** Muhammed Hanifi Bademkıran, Özcan Balat, Seyhun Sucu, Mehmet Obut, Hüseyin Çağlayan Özcan, Fatma Bahar Cebesoy

**Affiliations:** 1 Diyarbakır University of Health Sciences, Gazi Yaşargil Training and Research Hospital, Clinic of Obstetrics and Gynecology, Diyarbakır, Turkey; 2 Gaziantep University Faculty of Medicine, Department of Obstetrics and Gynecology, Gaziantep, Turkey; 3 Private Practise, Clinic of Obstetrics and Gynecology, Gaziantep, Turkey

**Keywords:** cffDNA, complete mole, gestational trophoblastic disease, partial mole, polymerase chain reaction

## Abstract

**Objective:**

To study cell-free DNA (cfDNA) levels in patients with gestational trophoblastic disease (GTD) in order to test the hypothesis that cfDNA circulating in maternal plasma could provide early detection of GTD.

**Materials and Methods:**

This study included 32 patients with GTD (complete mole and partial mole) and 30 non-GTD patients in the first trimester of pregnancy with no other medical problems. cfDNA levels in maternal serum were measured using polymerase chain reaction analysis on Y-chromosome–specific sequences.

**Results:**

cfDNA was found as 327±367 pg on average in the control group and 600±535 pg in the GTD group. Within the GTD group, the partial mole group had an cfDNA average of 636±549 pg, and the complete mole group had an cfDNA average of 563±536 pg. Although there was a statistically significant difference between the GTD group and the control group in terms of cfDNA (p=0.02), there was no statistically significant difference between the complete mole group and the partial mole group (p=0.76).

**Conclusion:**

Non-parametric analysis of covariance in terms of cfDNA in GTD was performed, thereby increasing its power and revealing a significant difference compared with the control group. This indicates that maternal peripheral bloodstream cfDNA monitoring might be significant in the early diagnosis of GTD.

**PRECIS:** cfDNA in gestational trophoblastic disease.

## Introduction

Gestational trophoblastic disease (GTD) refers to a spectrum of disorders that result from abnormal proliferation of the trophoblastic epithelium. These include premalignant disorders (hydatidiform moles) and malignant disorders (persistent/invasive gestational trophoblastic neoplasia, placental site trophoblastic tumors, epithelioid trophoblastic tumors, and choriocarcinoma). Most patients present with abnormal beta-hCG levels and abnormal ultrasound. Laboratory evaluation and imaging of the chest, abdomen, and pelvis are recommended to determine the appropriate treatment and follow-up. Complete recovery is possible in patients who are diagnosed early and who receive adequate treatment in the course of the disease^(1)^. Free DNA is naked, double-stranded DNA independent of the cell, and it can be obtained from the circulatory system by means of plasma purification or serum samples^(2)^. In the last 30 years, the importance of a detailed study of free DNA has become more apparent^(3,4)^. Approximately 95% of the free DNA isolated from the mother during pregnancy belongs to the mother, and 5% belongs to the fetus, and this is called cell-free DNA (cfDNA). It has been shown that 1 mL of blood in a mother with a healthy pregnancy usually contains one fetal cell; this ratio increases in the majority of pathologic pregnancies^(5,6)^. Circulating fetal DNA has also been identified as a marker for assessing fetomaternal well-being. Increased fetal DNA concentrations have so far been associated with a number of pregnancy-related complications, and serum-free fetal DNA elevation has been detected in conditions including pre-eclampsia, preterm labor, invasive placenta, hyperemesis gravidarum, fetal growth restriction, fetomaternal hemorrhage, polyhydramnios, and single gene disorders such as achondroplasia, myotonic dystrophy, congenital adrenal hyperplasia, beta thalassemia, cystic fibrosis, and Huntington disease(7,8,9,10,11,12,13,14,15,16,17,18,19). In the present study, we studied cfDNA levels in patients with GTD in order to test the hypothesis that cfDNA circulating in maternal plasma could provide the early detection of GTD.

## Materials and Methods

### Sample Collection

This study included 32 patients with GTD who were referred to Gaziantep University Faculty of Medicine, Department of Obstetrics and Gynecology, and 30 patients with first-trimester pregnancies with no medical problems. This study was reviewed by the appropriate ethics committee Gaziantep University (approval number: 05/2011-04) and was performed in accordance with the ethical standards described in an appropriate version of the 1975 Declaration of Helsinki, as revised in 2000. Preliminary diagnosis of GTD was made using ultrasound. All patients had a clinical assessment and transvaginal pelvic ultrasound performed by gynecologic sonographers working in the Early Pregnancy Unit (Toshiba XARIO-XG, Toshiba) of the hospital. If the uterus was enlarged, this was supplemented by a transabdominal approach. The ultrasound criteria for suspecting molar pregnancy were cystic changes, irregularity or increased echogenicity in the decidua, chorionic tissue or myometrium. The ultrasound criteria for suspecting malignant GTD were a hypoechoic or heterogeneous, predominantly solid tumor within the uterine cavity in the presence of a positive pregnancy test. In patients with this preliminary diagnosis, the uterine cavity was evacuated through revision dilatation and curettage. The evacuated material was sent for pathology analysis. Patients with histopathologically diagnosed GTD were identified using electronic patient records and Gaziantep University Faculty Medicine Hospital Trophoblastic disease records. The control group included first-trimester pregnancies with normal biochemistry and complete blood results without medical problems. Sociodemographic, reproductive, medical and laboratory data, fetal ultrasonographic information, and patient follow-up forms were collected from both groups. Control patients diagnosed as having GTD and any other medical problems were excluded from the study.

### DNA Extraction

Blood samples were collected into 12 mL EDTA collecting tubes from case patients and controls before evacuation or invasive procedures. The samples were sent to the laboratory within 15 minutes and centrifuged at 2480 rpm for 10 minutes and at 3600 rpm for 20 minutes. They were then kept in Eppendorf tubes at -80 °C. Working day materials were melted, and DNA absorbances were measured using spectrophotometry.

### Polymerase Chain Reaction Analysis

DNA isolation was performed on the first serum samples taken and in accordance with the protocol of the commercial company that provided the equipment (IONTEK MagCore, RBC Bioscience). The resulting DNA material was divided into two samples: one sample was used immediately at +4 °C and the other sample was stored (for subsequent repeats) at -20 °C. The purity of the DNA obtained was determined using spectrophotometry. DNA material isolated from the plasma of the pregnant women was subject to polymerase chain reaction (PCR) analysis specific to the Y-chromosomal sequence (DYS14). Following the same protocols for the serum taken from patients, dilutions of 1/10, 1/100, and 1/1000 were prepared. Sensitivity was designated as a dilution of 1/1000. The minimum DNA (Y-chromosomal) detection limit in the study was set at 10 pg, and the X-chromosomal detection limit was set at less than 10 pg. In order to demonstrate DNA isolation efficiency, PCR analysis specific to the human glyceraldehyde-3-phosphate dehydrogenase sequence was performed. Distilled water was used as a negative control.

### Statistical Analysis

Median and interquartile range are used in the presentation of numerical variables. Student’s t-test and the Mann-Whitney U test were used to compare continuous variables with and without normal distribution between the groups. Any differences were considered to be significant when the p value was less than 0.05.

### Outcome

This study evaluated cfDNA as the basis for the clinical outcome. The fixed variables were identified as common independent variables (covariates) between the GTD and control groups. Then, the difference between the fixed factor of the cfDNA was investigated.

### Candidate Covariates

It was crucial that the possible covariate taken as a model for GTD was clinically and biologically plausible and related to mole pregnancy in previous studies. These principles were used to determine the variables that were included in the model. Furthermore, gravida, parity, abortion, age, and weight were all identified as candidate covariates. Therefore, five candidate variables were included in the linear regression models.

### Sample Size Calculation

This study used analysis of covariance (ANCOVA) because the sample size was small. In ANCOVA, there is a fixed factor and a covariate, which is used to reduce the unexplained variation in the dependent variable, cfDNA, thereby increasing its power. The dependent variable, cfDNA, was divided into two error variances: variance resulting from a covariate and unexplained error variance. If we attribute this unexplained variance to a covariate, we can reduce the unexplained error variance. Therefore, the variance in the dependent variable (i.e., the cfDNA group) is explained by the fixed factor, as well as the error variance and variance arising from the groups. In this way, the unexplained variance is reduced.

### Statistical Modelling

The interaction between GTD and the control group as the fixed factor with the candidate covariates was used to form a linear regression model with a dependent variable, cfDNA. Regression modelling strategies and special transformation functions were applied to the non-standard distribution variables in the model. The residual variables were determined and ANOVA was performed after creating the linear regression models.

### ANCOVA Performance and Validation

A standard calibration curve was drawn for all groups to represent the general relationship between cfDNA and the candidate covariates. This calibration curve is presented as a scatter plot in the lower panel of the correlogram matrix. The middle panel presents the distribution plots of the variables in the histogram, and the upper panels show the correlation coefficient of the variables. All statistical analyses were explained using the R (R Statistical Software, Institute for Statistics and Mathematics, Vienna, Austria) software package version 3.5.1.

## Results

**Figure 1:**
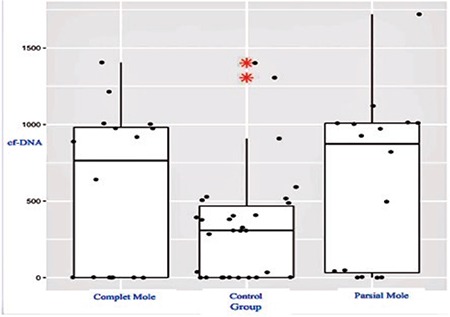
Plot of complete and partial mole groups according to a comparison of the cfDNA and control groups

**Figure 2:**
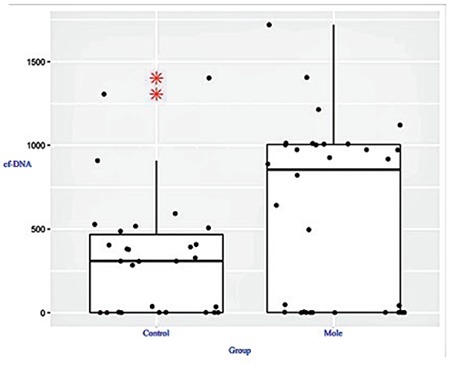
Plot of the GTD group according to a comparison of the cfDNA and control groups

**Figure 3:**
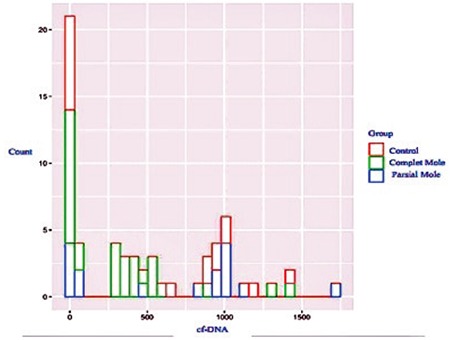
Plot of complete and partial mole groups according to a comparison of the cfDNA and control groups

**Figure 4:**
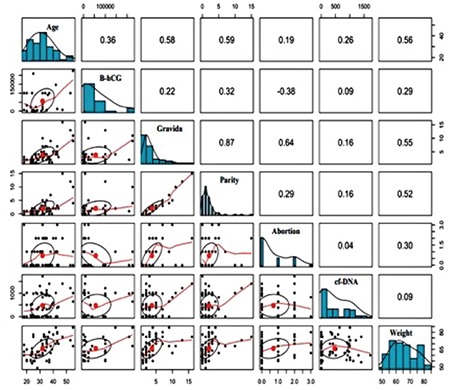
The lower panel of the matrix shows a scatter plot of all variables and provides the homogeneity of the calibration curves. The middle panel of the matrix shows the histogram distribution plots of the variables, and the upper panel shows the correlation coefficient

This study included 62 patients. Of the 32 patients diagnosed as having GTD, 16 were reported as complete moles and 16 were reported as partial moles. The control group consisted of 30 pregnant women who were healthy during the 12^th ^gestational week. Tables 1 and 2 summarize the baseline clinical characteristics of the study groups. A statistically significant difference was observed between the GTD and control groups in terms of cfDNA (p=0.02) (Table 1), but there was no statistically significant difference between the complete and partial mole groups (p=0.71) (Table 2). Figures 1, 2, and 3 compare the cfDNA parameters for the groups. In terms of cfDNA as dependent variable made ANCOVA. Moreover, the participating candidate covariates showed a significant difference when compared with the fixed factor (Table 1). The performance and validation of the variables using ANCOVA are presented in the correlogram matrix (Figure 4).

## Discussion

This study measured the increase in cfDNA levels in GTD in order to test the hypothesis that cfDNA circulating in maternal plasma could provide early detection of GTD. Non-parametric ANCOVA in terms of cfDNA in GTD was performed, thereby increasing its power and revealing a significant difference compared with the control group. Tjoa et al.^(20)^ found an increase in free fetal-placental DNA in maternal serum due to trophoblastic oxidative stress, resulting in trophoblastic degeneration. Therefore, concentrations of cfDNA in maternal serum and plasma may act as a biomarker of trophoblast well-being during pregnancy, and could provide a scientific rationale for the administration of antioxidant vitamins in high-risk pregnancies. Alberry et al.^(21)^ showed that cfDNA was the primary source of maternal plasma, which supports the hypothesis that trophoblasts are a source of cfDNA in maternal plasma in anembryonic pregnancies. These two studies indicated that trophoblastic injury might cause the level of cfDNA to increase in maternal serum, and our study showed high levels of cfDNA in GTD.

Sifakis et al.^(22)^ found that the concentration of maternal cfDNA increased at 11-13 weeks of gestation in pregnancies that experienced early-onset preeclampsia. This provides further support for the presence of impaired placentation in the pathogenesis of the disease. Yin et al.^(23) ^found that concentrations of maternal plasma fetal DNA and total DNA increased throughout the first trimester. Significantly high levels of fetal and total DNA were also found in pregnancies that miscarried. The integration of non-invasive prenatal diagnosis into clinical care has identified new aspects of perinatal biology, mainly cfDNA, which is a new biomarker that can provide information about the placenta and can potentially be used to predict clinical problems^(24)^. Openshaw et al.^(25)^ showed that cfDNA could be detected in the plasma of women with trophoblastic tumors and facilitate diagnosis. Although cfDNA could be used as a marker for identifying subjects at increased risk of developing pre-eclampsia, it is not suitable for measuring cfDNA levels to diagnose ectopic pregnancies in the early period^(26,27)^. The present study shows that the cfDNA increase is significant during the early period of GTD. One of the limitations of our study is that the cfDNA-DYS14 assay method was used to isolate cfDNA from the mother’s plasma. This is the oldest method and does not work with new methods of study. We used this method because it was the most cost-effective given that the authors themselves met the cost of financing the tests.

## Conclusion

All aspects of the pathophysiology of GTD have not yet been elucidated. Therefore, it can still lead to morbidity and mortality. In order to reduce these risks, it is necessary to focus on practical and cost-effective solutions, especially ones that are likely to assist with early diagnosis. Few studies have investigated the relationship between GTD and cfDNA; however, this is likely to change in the coming years as cfDNA becomes recognized as an essential focus of research. Based on existing studies, we can predict that maternal cfDNA levels increase due to placental damage in pathologic pregnancies. This study measured cfDNA using PCR analysis of maternal serum, and the results show significant differences between the patient and control groups. We also suggest that monitoring the maternal peripheral bloodstream for cfDNA could be significant for the early diagnosis of GTD.

## Figures and Tables

**Table 1:**
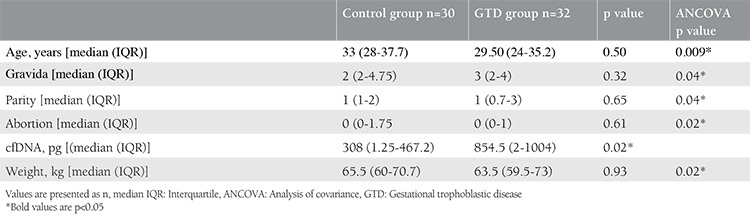
General characteristics used to compare the control and gestational trophoblastic disease groups

**Table 2:**
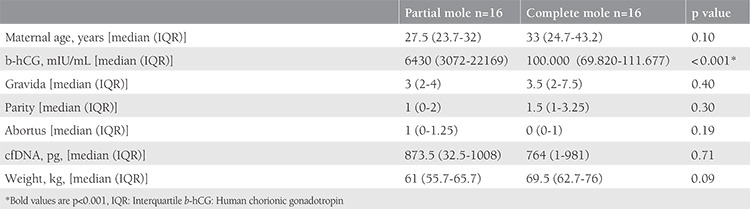
Statistical parameters used to compare the complete and partial mole groups
